# Peer support as pressure ulcer prevention strategy in special school learners with paraplegia

**DOI:** 10.4102/sajp.v80i1.2047

**Published:** 2024-07-30

**Authors:** Undine S. Rauter, Desmond Mathye

**Affiliations:** 1 Department of Physiotherapy, School of Health Sciences, Sefako Makgatho Health Sciences University, Pretoria, South Africa

**Keywords:** adolescents with paraplegia, pressure ulcer prevention, support group, peer mentoring, physiotherapist’s role

## Abstract

**Background:**

Adults with spinal cord injuries perceived peer support as beneficial in preventing secondary health conditions, but the role of peer support among adolescent learners with paraplegia in special schools is still unknown.

**Objectives:**

To explore the perspectives of current and previous learners with paraplegia on peer support to prevent pressure ulcers in a special school.

**Method:**

A qualitative, exploratory, descriptive study design was used. The authors conducted 12 semi-structured telephonic, audio-recorded interviews and a focus group discussion with current and previous learners with paraplegia attending a special school. The interviews were transcribed verbatim and translated, and data were organised using the Nvivo-12 Pro program. Through conducting an inductive thematic analysis categories, sub-themes and themes were identified from the participants’ narratives.

**Results:**

The participants’ perspectives included positive and challenging aspects of peer support reflected in four sub-themes: group-based support, individual peer mentoring, challenges with peer support and the roles of the school physiotherapist regarding peer support.

**Conclusion:**

Learners saw peer support as a crucial strategy in preventing and reducing pressure ulcers. Establishing a peer support system with group and individual components in special schools could be a game-changer to end pressure ulcers among learners with paraplegia and ensure better health and educational outcomes.

**Clinical implications:**

Physiotherapists in special schools should support peer support initiatives among learners with paraplegia to ensure successful pressure ulcer prevention.

## Introduction

Spinal cord injury (SCI) can manifest as paraplegia, which significantly impacts an individual’s life and social network. This condition, resulting from either traumatic or non-traumatic causes, brings about profound functional impairments. According to Guest, Fuller and Vowden (2022), these impairments include loss or reduction of active movement, mobility, sensation and circulation, as well as control over bowel and bladder functions, alongside difficulties with sexual functioning. Furthermore, secondary health complications such as pain, spasticity, contractures and pressure ulcers are common (Pilusa, Myezwa & Potterton 2021a; Vicen & Gater 2022). Among these complications, pressure ulcers are notably severe and impactful. Gefen et al. (2022) describe pressure ulcers as injuries to the skin and underlying tissues, primarily around bony prominences. These injuries result from pressure, friction and shear forces at the support surface or a combination of these factors. The global prevalence of pressure ulcers among SCI patients is approximately 32.36%, with a higher prevalence of 41.19% in Africa (Shiferaw et al. 2020). The classification system for pressure ulcers, as outlined by the EPUAP/NPIAP/PPPIA pressure injury alliance in 2019, delineates four stages based on the depth of tissue breakdown, ranging from reddening to deep wounds that may involve muscles and bones.

In addition, two stages are designated for unstageable or suspected deep tissue injuries.

The progression of pressure ulcers can lead to extended hospital stays, and an increase in morbidity and mortality rates, particularly in South Africa, where studies by Mashola, Olorunju and Mothabeng (2019), along with Madasa, Boggenpoel and Phillips (2020), highlighted the grave implications of such complications.

The risk of developing pressure ulcers is influenced by a complex interplay of global health, care, biomedical and biomechanical factors (Cao, DiPiro & Krause 2019; Mervis & Phillips 2019). While much of the existing research has focused on adults, children and adolescents with SCI, including those with congenital spinal cord defects or acquired SCI before their growth spurt, face unique risks. These include spinal asymmetry and/or scoliosis and the risk of hip dislocation because of underdeveloped trunk and hip muscles, leading to asymmetric pressure distribution and an increased risk of pressure ulcers, especially around the ischial tuberosity (Zepou, Benetos & Pneumaticos 2022). Moreover, children’s distinct self-care abilities and daily activities, such as playing on the floor, can pose specific risks for pressure ulcers on the knees (Zebracki et al. 2020).

Prevention strategies commonly include the use of pressure-reducing surfaces, such as wheelchair cushions, and the implementation of both active and passive pressure relief techniques (McNichol et al. 2015). Appropriate wheelchair and seating provision, good nutrition and the control of spasticity and incontinence are also crucial (Stevens & Bartley 2018). For children and adolescents, these prevention strategies must be adapted to their developmental stage, encouraging them to progressively assume responsibility for their self-care, including pressure ulcer prevention during play. This necessitates additional support and caregiver engagement, although the involvement of multiple caregivers, as seen in special schools, can complicate the consistent application of these strategies (Park et al. 2014).

Despite the severe consequences of pressure ulcers, including high risks of relapse, social isolation and increased healthcare costs, there is a notable gap in prevention systems within special schools, as observed through multiple admissions for severe pressure ulcers in a district hospital over the last 15 years (Pilusa et al. 2021a). This underscores the need for further exploration of this issue among children and adolescents in South African special schools.

The role of paraplegic peer support in preventing and managing the secondary health complications of SCI has been gaining recognition, with positive outcomes reported in high-income countries such as Canada, Denmark and Australia, through various forms of peer support services (Barclay & Hilton 2019; Best et al. 2023; Divanoglou, Tasiemski & Jörgensen 2020; Hoffmann, Sundby & Biering-Sørensen 2019). Such initiatives have been linked to improvements in self-efficacy, prevention and management of pressure ulcers and overall quality of life for individuals with SCI. Despite these advancements, the need for peer support remains significantly unmet in low- and middle-income countries, including in Asia and Africa, where peer support has been suggested as an effective tool for preventing secondary health complications (Abedi et al. 2022; Pilusa, Myezwa & Potterton 2022; Trezzini et al. 2019).

In Southern Africa, some peer support initiatives, such as those by the NPO ‘Siletha Ithemba’ in rural KwaZulu-Natal (KZN), South Africa, and the peer-led ‘Active Rehabilitation’ block training in Botswana, demonstrate the potential benefits of peer counselling, wheelchair training and advice for community members with SCI (Divanoglou et al. 2019; Salojee & Bezuidenhout 2020). However, these programmes primarily focus on adults, with limited research on peer support for learners with paraplegia in South African special schools. While studies on peer support among able-bodied learners in Southern Africa exist (Chinyama, Rembe & Sibanda 2020; Mitchell 2023), and a recent study by Harrison et al. (2023) highlights the benefits of clinic-based peer support groups for adolescents with mixed chronic illnesses, the specific impact of peer support for learners with paraplegia remains to be fully explored.

### Theoretical framework

Our study’s theoretical framework integrates Bronfenbrenner’s bio-ecological systems theory (Samuels, Stemela & Booi 2020) with the International Classification of Functioning, Disability and Health Framework (ICF). These comprehensive approaches provide a multilayered perspective on child development, disability and rehabilitation within the context of community and societal structures.

At the core of our theoretical framework is Bronfenbrenner’s bio-ecological systems theory, which situates the child within a complex network of relationships spanning familial, educational and broader community contexts. This theory underscores the significance of dynamic interactions between the child and their immediate and extended environments, emphasising how these reciprocal relationships shape the child’s development and experiences (Samuels et al. 2020).

Building on this, the International Classification of Functioning, Disability and Health Framework (ICF) offers a model for understanding the multifaceted nature of disability (WHO 2013). It delineates how individuals’ internal conditions interact with various environmental and societal factors, presenting a holistic view of the disability’s implications for an individual’s daily functioning and overall well-being.

The focal point of our study is the exploration of learner’s interactions within the school environment, with a special emphasis on how peer relationships affect their health and well-being. Through the lens of the aforementioned theoretical frameworks, our study aimed to uncover the complex dynamics at play within educational settings that influence the well-being of children with disabilities. This integrated theoretical approach facilitates a deeper understanding of educational experiences and offers strategic insights for fostering supportive, healthy and inclusive environments for all children.

### Aims and objectives

This article reports on one objective of a larger PhD study that aimed to develop a model for preventing pressure ulcers in a South African special school. Our study objective was to describe the experiences of current and previous learners with paraplegia with pressure ulcers during their school attendance and explore their ideas to improve pressure ulcer prevention in the special school, resulting in a prevention model. The participants suggested peer support as a crucial pressure ulcer prevention strategy, which was then explored. The evolving perspectives of previous and current learners on peer support are reported in this article.

## Research methods and design

### Study design

Our study adopted a qualitative, exploratory and descriptive approach to examine and report on a phenomenon that has received limited research attention, focusing on the participants’ perspectives regarding an issue of concern to them. By employing this approach, our study amplified the voices of the participants, allowing them to articulate their proposed solutions to the challenge of preventing pressure ulcers. It then situated these participant-generated solutions within the broader context of existing research evidence, offering a nuanced discussion that bridges individual experiences with scholarly findings (Salmiranta et al. 2024).

### Setting

Our research was conducted in a public boarding school for students with physical disabilities in the rural Ngaka Modiri Molema District of the North West province, South Africa. This special school included in our study caters to approximately 180–190 learners aged between 6 years and 27 years, spanning grade 1 to grade 12. The student body comprises learners with a variety of physical disabilities including cerebral palsy, amputations, muscular dystrophy and other mobility impairments. The data collection phase coincided with coronavirus disease 2019 (COVID-19) pandemic restrictions, necessitating telephonic interviews with participants who, because of the lockdown, were in their homes across different locations within the North West province.

### Study population and sampling strategy

Our study focused on current and former learners from a specific special school, with a particular interest in those who have paraplegia and experienced pressure ulcers. The population of interest was derived from approximately 18% of the school’s enrolled learners who had paraplegia.

#### Inclusion criteria

Eligible participants for our study were:

- Current learners (CLs) or previous learners (PLs) of the selected special school
- Individuals with paraplegia who had experienced pressure ulcers
- The participants were aged between 18 years and 30 years accommodating both current learners and those who had attended the school up to 7 years back.

This age criterion was selected to ensure participants were capable of articulately sharing and reflecting on their experiences.

#### Sampling strategy

A purposive sampling method was used, targeting individuals who met the inclusion criteria relevant to our study’s objectives. This approach facilitated the exploration of the research topic through the perspectives of participants possessing characteristics pertinent to the research goals.

### Sample size

The sample consisted of 12 individuals with paraplegia: six current and six PLs from the school. Initial contact was made with 27 potential participants through the assistance of a staff member (15 CLs and 12 PLs), out of which 13 agreed and provided informed consent. Ten were excluded for not meeting the inclusion criteria and four declined to participate. One underage participant, 16 years old, was included with the guardian’s consent and the participant’s assent. However, one interview was disregarded because of technical issues, resulting in a final sample size of 12 participants, as acceptable in qualitative research (McMillan & Schumacher 2006; Mocănașu 2020). The participants’ anonymity was maintained by removing identifying characteristics and remarks from narratives in reports of our study.

### Data collection

Demographic data were gathered using a self-designed data collection sheet, which the first author administered during a telephonic conversation with the participants. At the end of the session, a convenient date and time for the participant was determined for the main interview. The first author held 12 semi-structured, audio-recorded interviews between August 2020 and March 2021 in the participants’ preferred language. The conversations followed an interview guide, containing five basic questions about the participants’ experiences with pressure ulcers, how they affected them, perceived causes, barriers to prevention and how prevention could become more effective. Most responses around peer support evolved in response to the question how to improve pressure ulcer prevention. The majority of participants chose to answer in the predominant local language of the North West province, Setswana, which the interviewer was fully proficient in (STATS SA, Census 2022). A focus group discussion with four participants who had also been individually interviewed served as a member check and to deepen specific topics through discussions among participants (Thomas 2017; Zairul 2021).

### Data analysis

All interviews were audio-recorded, immediately transcribed verbatim, translated into English and the data were organised using the Nvivo-12 Pro program for qualitative research. The English translation was verified through a language quality review by the Setswana language department of the North West University. The first author conducted an inductive thematic analysis by coding the interview narratives line by line. Similar codes were aggregated into categories, from which subthemes and themes emerged (Braun & Clarke 2012). A reflexive journal, mind-maps, discussion with the second author, and a literature review supported the analytic process (McMillan & Schumacher 2010).

### Trustworthiness

Our study sought to adhere to most of Lincoln and Guba’s four criteria for trustworthiness – credibility, transferability, dependability and confirmability to ensure the integrity and reliability of its findings (Alexander 2019). The foundation for establishing credibility was the authors’ extensive experience, exceeding 25 years, in a district hospital adjacent to our study area, coupled with a deep understanding of the participants’ culture and language. To mitigate investigator bias and bolster credibility, our study employed strategies such as direct quotations from participants and member checking, where participants reviewed the accuracy of how their experiences were represented. Furthermore, to enhance credibility, the authors engaged in ongoing self-reflection, meticulously documented in a reflexive journal. This reflexive practice and discussions on the research process and findings with the co-author provided an additional layer of scrutiny and introspection.

To show confirmability, recognised research methods and a detailed exposition of these methods and the analytical process were used. Methodological transparency and member checking underpinned the confirmability of the research, ensuring that findings were well founded and impartially reported. However, the findings of our study are only transferable to contexts similar to those of our study. According to Korstjens and Moser (2018), diligent adherence to established research practices and methodologies is critical for affirming the trustworthiness.

### Ethical considerations

Our study was approved by the Sefako Makgatho Health Sciences University’s Research and Ethics Committee (no. SMUREC/H/36/2020: PG) as part of a larger study for a PhD first from June 2020 with continuation until December 2023. The necessary permissions of all involved departments, institutions and individuals in our study were obtained. Participants were protected against any possible harm in connection with our study, and they did not derive any direct benefit from participating in our study (World Medical Association 2013). Considering the health and safety of the participants, possible exposure to the COVID-19 SARS virus in conjunction with our study was eliminated by using exclusively virtual data collection methods (Williams 2008). While telephonic data collection has the disadvantage of missing clues from participants’ body language, it can reduce bias by concealing the non-verbal reactions of the interviewer to the participants (Saarijärvi & Bratt 2021). Access to the data was limited to the authors (Kirilova & Karcher 2017). Confidentiality of the data was ensured by allocating participant codes and removing any identifying characters such as references to names or locations in the narratives.

Participants received an information letter and consent form in their preferred language (either English or Setswana) using WhatsApp messages or emails. The authors explained the content during a telephone call. All participants provided voluntary, written informed consent. In the case of the minor, caregiver consent and participant assent were obtained.

## Results

### Demographic characteristics of participants

Of the 12 participating learners with paraplegia, 58% (7) were female participants. Fifty-eight per cent had acquired paraplegia from traumatic (4) and non-traumatic causes (3), and 42% (5) had congenital spinal cord dysfunction (spina bifida). Half of the participants reported severe pressure ulcers of stage 4 or unstageable during their school attendance, while 42% (5) reported stage 2–3 PU. One learner had not experienced any pressure ulcer beyond stage 1. At the time of the interview, only two learners still had pressure ulcers.

Half of the participants were in the age group of 22 years – 27 years, with the mean age of the CLs being 20.7 years, while the mean age of PLs was 24.4 years. Current learners attended grade 5 to grade 9, while all PLs had completed grade 12 up to 7 years back. Among the PLs, two were employed or self-employed, two were enrolled in studies, and two were unemployed. Seventy-five per cent of the participants stayed in rural villages during their school attendance. The participants’ individual characteristics are depicted in Table 1.

**TABLE 1:** Demographic characteristics of learners with paraplegia (U. Rauter/2024).

Participant	Gender	Age (years)	Home residence	Academic achievement	Present occupation	Diagnosis	Experienced PU stage
CL 1	M	22	Rural Village	Attends Gr 8	Learner at school	TSCI	4†
CL 2	M	18	Rural Village	Attends Gr 7	Learner	SB	2–3
CL 3	M	19	Rural Village	Attends Gr 9	Learner	TSCI	4 or unstageable
CL 4	F	16	Semi-rural Village	Attends Gr 5	Learner	SB	2-3
CL 5	F	27	Rural Village	Attends Gr 8	Learner	TB spine	1 or 0
CL 6	F	22	Rural Village	Attends Gr 7	Learner	SB	2–3
PL 1	M	27	Rural Village	Gr 12	Unemployed/no study	SB	4
PL 2	F	23	Rural Village	Gr 12	TVET student	TB spine	4†
PL 3	F	26	Rural Village	Diploma	Student	TSCI	4†
PL 4	F	21	Rural Village	Gr 12	Unemployed/no study	TB spine	2–3
PL 5	F	24	Township	Diploma	Learnership	SB	2–3†
PL 6	M	30	Township	NQ 5 Certificate	Self employed	TSCI	2–3

TSCI, Traumatic spinal cord injury; SB, Spina bifida; TB spine, Tuberculosis of the spine; F, female; M, male; TVET, Technical and Vocational Education and Training College; CL, current learner; PL, previous learner.

† , multiple.

### Results from the content analysis

In response to the question of how to improve pressure ulcer prevention in the school, participants suggested peer support as a valuable strategy to prevent and combat pressure ulcers. When exploring this theme, four sub-themes arose from the participants’ narratives: (1) group-based peer support, (2) individual peer support, (3) challenges with peer support and (4) the role of the school physiotherapist regarding peer support. While most quotations reflected in the following paragraphs are taken from PLs because of their better ability to express themselves, CLs expressed similar sentiments. In the next sections, each sub-theme and its categories are presented in an introductory summary, followed by quotations. The terms in parenthesis in the beginning of the quotation indicate the applicable codes.

#### Sub-theme 1: Group-based peer support

Participants suggested establishing a paraplegic support group in which learners with paraplegia would regularly meet. Three categories further illustrated the participants’ perspectives: (1) purpose of the peer support group, (2) organisational considerations and content of the support group and (3) the leadership of the support group.

**Purpose of the peer support group:** The participants saw the peer support group as a safe space for mutual support, helping them to freely express themselves and learn to handle various aspects of their lives more successfully. In addition, teaching one another crucial self-management competencies, role modelling, peer monitoring and encouragement or counselling to initiate behavioural change and self-representation/advocacy were essential functions of the group. Previous learners were concerned about the next generations of learners, which they thought particularly needed support and guidance from the group. The PLs also hoped group members would transfer learned skills and behaviours into everyday life in the school. As a result, they expected pressure ulcers to decrease and ultimately disappear in the school:

‘[*Concern for next generation*] … More especially concerning those who come … behind us they are not the same as we.’ (PL2, female, 23 years)

‘Cause the generation after me, all of them use pampers. They don’t use the catheters.’ (PL3, female, 26 years)

‘[*Safe space*] … so that they can talk to a person whom they can trust … We will be more open … we discuss our issues – that we can express ourselves … we share our problems.’ (PL1, male, 27 years)

‘[*Support/ Solidarity*] … especially things like pressure sores: A normal person cannot understand, what we went through.’ (PL2, female, 23 years)

‘[*Teaching one another*] … if there would be a plan … that those children who have it and those who haven’t a pressure sore, would be taught ….’ (CL1, male, 22 years)

‘Maybe in form of sessions … teach the younger ones in the end and the new ones, who couldn’t go to rehab.’ (PL6, male, 30 years)

‘[*Self-management*] Cause … all of them use pampers. They don’t use the catheters.’ (PL3, female, 26 years)

‘[*Alerting peers*] … at the support group, we can raise a point not directly to that particular person, but indirectly.’ (PL6, male, 30 years)

‘[*Peer monitoring*] And at other times to know one another and to be seen: You are made aware by another person saying: look, it is like this and that with you, so please go to the clinic and seek some help … to be told by another person “deal like this with your life.”’ (PL5, female, 24 years)

‘[*Advocacy*] … if one or two persons could go, and they represent the paraplegics, and they would talk with someone from physio.’ (PL3, female, 26 years)

‘[*Prevent / combat pressure ulcers*] I feel if this could happen in this way, most people would not go through these stages that they would sit with the pressure sores. … They wouldn’t have to pass through where we have been passing through … there would be the ability to finish with the pressure sores.’ (PL2, female, 23 years)

**Organisational considerations and content of support group:** Predominantly PLs suggested the support group should target learners with paraplegia, including those with spina bifida, with and without pressure ulcers, and meet weekly on a specific day under the leadership of a peer supporter/mentor:

‘[*Forming a group*] I would open something like a Stokvel, something like group.’ (CL3, male, 19 years)

‘[*Target group*] If it was me who is in charge of the school, I would form maybe a group of paraplegics and spina bifidas … Then, it would be like that we meet. … And it could be better if they would have a para group.’ (PL3, female, 26 years)

‘[*Regular meetings*] In the special schools, there should be every week, should be some sort of a group … They should have a group, a group for themselves … for example on Wednesdays.’ (PL2, female, 23 years)

Each meeting should include input around a paraplegia-related topic, such as the condition itself, pressure ulcers, pressure ulcer prevention strategies, care products, hygiene and other relevant self-management practices. Furthermore, free discussions, sharing tips and tricks and addressing learners’ behaviours would be part of the group sessions:

‘[*Pressure ulcer risk and development*] And we would talk about pressure sores: What is the risk? How do they develop in you?’ (CL3, male, 19 years)

‘ … they would be taught about pressure sores. If they would be taught about the cruelty of this condition, how it is, and about its cruelty.’ (CL1, male, 22 years)

‘[*Pressure care*] we would talk about pressure care … then they teach them the pressure what what … They will talk about the suggestions, “guys, we’ll do this [*to*] relieve pressure.”’ (PL3, female, 26 years)

‘[*Self-management and Hygiene*] … How to live being clean, how to take care of yourself.’ (PL3, male, 27 years)

‘ … will be able to teach the younger ones in the end and the new ones, … this self-management.’ (PL6, male, 30 years)

‘[*Skin care*] … and also the lotions, there are some people who say, “I apply this lotion at the skin. My skin is not too dry, so how about you try this lotion … ?”’ (PL3, female, 26 years)

**The leadership of the support group:** Participants envisioned the group being led by a mature, knowledgeable peer with paraplegia who had been through the experience and could be a role model for the group members regarding preventing and overcoming pressure ulcers. The group leader would be expected to facilitate trust and openness of the group members through good communication skills:

‘[*Been through it*] It is important, because he or she would be more experienced … that he or she already has passed, which we still would experience. … each and every time, he or she will advise us, you will come across this and that.’ (CL1, male, 22 years)

‘[*Role model*] The group leader could be someone who knows pressure sores … The person could be a good example because they know him. They saw how this condition of pressure sores had made him struggle. So, if he would explain to them saying “so guys, I’m here because of pressure sores” … they too will start to take care of themselves.’ (PL3, female, 26 years)

‘[*Approachable, confidentiality*] … someone, who is more open. We will be more open, being free towards him or her. It shouldn’t be a person who, when we meet her or him, and we talk about life, we are afraid to confide in them … if we talk, this should stay between us.’ (PL1, male, 27 years)

‘[*Non-judgemental*] … he or she can understand the reasons or motivations of them … what happens.’ (PL1, male, 27 years)

‘[*Good listener*] He or she should listen attentively to them, and be there for them.’ (PL1, male, 27 years)

#### Sub-theme 2: Individual peer support

Three categories illustrated aspects of individual peer support: (1) characteristics and benefits of individual mentoring, (2) being a peer supporter and (3) the role of friends.

**Characteristics and benefits of individual peer mentoring:** A CL described the benefits of mentorship by an elder peer similarly to the benefits of group support: being understood, emotionally supported and having a role-model to relate to on a personal level. Receiving specific guidance and support based on knowing one’s individual background was rated positively:

‘[*Benefits of personal mentoring*] … he usually comes, and comforts me. And he advises me where he sees, that I get lost on the way … On my journey, he is the one who understands me … my situation, … my background. I took him like my brother …. my role model. I follow his steps.’ (CL1, male, 22 years)

**Being a peer supporter:** Some PLs indicated that they were informal peer supporters to younger CLs at the school. They saw their role in answering questions and giving younger learners practical tips through telephonic engagements. Furthermore, some PLs also tried to give advice to peers in other contexts:

‘[*Peer support by PLs to CLs*] And there are some who I am close with. They are able to be open to me. I still have their contacts. They can tell me: “ijoo, I have a pressure sore, how should I handle it?” I say “go there” or “you know, I have something red how should I handle it?”’ (PL3, female, 26 years)

**The role of friends:** Friends played a dual role. Some friends were described as supportive and encouraging self-care, while others were seen as distracting learners with paraplegia from adhering to health routines. In the following paragraph, some challenges with peer support are reported.

#### Sub-theme 3: Challenges with peer support

Three categories illustrated the learners’ challenges with peer support: (1) a lack of support by friends, (2) peer pressure and (3) the rejection of peer support offered by PLs.

**A lack of support by friends:** Some participants described their disappointment with their friends, which could also include peers with other disabilities. They classified some of their peers as ‘fake friends’ because they expected true friends to support them in adhering to necessary health protocols instead of pulling them down:

‘[*Fake friends*] … you can see, you got fake love, fake friends, they are many and useless.’ (CL3, male, 19 years)

‘When you need something from your friend, they never help you, they just look at you and leave.’ (CL4, female, 16 years)

‘[*Being pulled down*] … there are friends who turn you down … There is a person of who you think, he couldn’t destroy your life, because you live with them.’ (PL1, male, 27 years)

**Peer pressure:** In hindsight, PL perceived peer pressure as a challenge. In their quest to fit in, learners with paraplegia compared themselves to others with different disabilities and compromised their health care because they were afraid of discrimination by their peers:

‘[*Comparing to others*] You compare yourself with a person who doesn’t use a wheelchair and who is not in this position.’ (P2, female, 23 years)

‘[*Fear of discrimination*] … because that is why in the end we hide because we are afraid of other disabilities. If they could know that we have wounds, jioo! it will be another story.’ (PL6, male, 30 years)

‘… some of the things, which were incapacitating me, was that matter of “if I do this, what will my friends say?”’ (P1, male, 27 years)

**Rejection of peer support**: Previous learners who tried to provide peer support in other contexts were discouraged by negative reactions when their peers questioned their authority and rejected their advice:

‘[*Rejection of peer advice*] When you talk to the person and you tell her or him that maybe your problem is this, he or she will answer you like: Hey, you, you are not a doctor, since when do you know these things?’ (PL3, female, 26 years)

‘[*Discouragement*] … You think you help him or her out of love or out of knowing: I have been in that situation. I have at least a little bit of knowledge about this matter, but then he or she asks you such questions’ (PL3, female, 26 years)

#### Sub-theme 4: Physiotherapists’ role regarding peer support

Previous learners with paraplegia expected the school’s physiotherapist to provide guidance to the peer-group leader by being an approachable source of knowledge, teaching peer supporters, assisting with occasionally giving input and receiving feedback from the group sessions. In addition, they hoped physiotherapists would advocate for their needs at the school management level:

‘[*Assistance through physiotherapists*] … there should be the likes of … the help of the hospital physiotherapist, and the school, and other special schools, we will manage.’ (PL2, female, 23 years)

‘[*Feedback to physiotherapist*] If one person could go or two persons, and they represent the paraplegics, and they would talk with someone from physio.’ (PL3, female, 26 years)

‘[*Advocacy by physiotherapist*] They should raise this by someone … maybe, the physio-people.’ (PL3, female, 26 years)

## Discussion

Our study aligns with previous research both locally in South Africa and internationally, demonstrating the significant value individuals with SCI place on knowledge and advice from their peers who share similar experiences, which highlights the credibility given to peer-shared insights (Haas, Prince & Freeman 2013; Njoki, Frantz & Mpofu 2007). Pilusa, Myezwa and Potterton (2021b) further advocated for interventions focusing on self-management strategies that empower individuals with SCI to proactively manage their health and mitigate Secondary Health Conditions (SHC). These strategies, however, vary across different age groups.

In South Africa, youth friendly health services that incorporate peer support are gradually being introduced. Yet, they lack specificity towards disabilities and are absent in special schools (James, Pisa & Imrie 2018). Munce et al. (2014) conducted a scoping review that revealed a preference among adults with SCI for individualised peer support forms, such as peer mentoring and tele-support. Contrarily, adolescents from the school participating in our study reported no formal engagement with these support systems, finding face-to-face interactions more beneficial for fostering a trustworthy environment conducive to learning and behavioural change initiation.

Reflecting on their experiences, former students particularly emphasised the importance of peer support within the educational setting for preventing neglect or denial of necessary self-care practices, including pressure ulcer prevention. According to Rocchi et al. (2021), the significance of peer support in enhancing resilience and promoting self-management was also highlighted in community-based SCI peer mentorship programmes in Canada. However, a noticeable gap exists in the special school context, where there is a strong need for structured peer support. Previous students attempted to fill this gap through informal, relationship-based support. Still, this approach was often limited and sporadically dismissed outside their immediate circle, indicating a need for a more formalised structure.

Research from Canada underscores the need to train and coach peer supporters to ensure effective support systems (Martin Ginis et al. 2028). Similarly, Gainforth et al. (2019) suggested that such training should aim to equip peer supporters with the necessary knowledge and skills to meet learners’ expectations for maturity, role modelling and confidentiality. Chinyama et al. (2021) also advocate for ongoing mentoring and training of peer supporters in South African schools to maintain high-quality peer support.

Our study’s participants preferred physiotherapists to lead professional guidance, given their expertise and capacity to support a structured paraplegic peer support system. Through coaching and occasional direct involvement, physiotherapists could play a critical role in establishing a robust peer support system. Based on findings by Manamela et al. (2021), the potential of physiotherapists to advocate for and empower students with disabilities within the school system can significantly contribute to integrating peer support into the school’s health services.

Ultimately, a comprehensive peer support system, underpinned by strong professional and administrative support, could significantly improve self-management abilities among learners, including effective pressure ulcer prevention. Incorporating peer support into a holistic, youth-focused health approach could transform the prevention and management of severe pressure ulcers in special schools, marking a significant advancement in care and support for students with SCI.

### Strengths and limitations

To the knowledge of the authors, their study is the first of its kind in South Africa that explores perspectives of learners with paraplegia on peer support and its relevance for pressure ulcer prevention in the special school setting. Another strength of the study was the predominant use of the participants’ home language, which facilitated the participants’ openness to share their experiences and feelings.

However, the study also had limitations. Data collection was limited to non-contact methods because of the COVID-19-related lockdown restrictions, excluding field observations and non-verbal cues from body language from the analysis as a result of the telephonic interviewing. On the other hand, telephonic interviews can yield a similar quality of data and prevent interviewer bias (Kee & Schrock 2020; Rahman 2015). Furthermore, with only one participating school and a relatively small sample size, the study’s results may not be generalised to all boarding schools for learners with physical disabilities and across provinces – although they might face similar challenges and are governed by similar policies. The findings are not applicable to day scholars attending mainstream schools.

### Clinical implications and recommendations

Peer support can become a catalyst for improving health outcomes of learners with paraplegia in special schools. Trained previous and current learners with paraplegia could assume peer mentoring roles to CLs and lead support groups, based on their experiences in the setting. If peer support would be formalised, peer supporters could become instrumental to improving health outcomes of learners with paraplegia. To harness the full potential of peer support for pressure ulcer prevention in special schools, collaboration between peer supporters and a health professional with the appropriate skills, knowledge and proximity to the learners is beneficiary. Such collaboration would enhance the learners’ self-management competencies with a ripple effect on their educational experience through improved health. The learned life skills would be applicable even beyond the school environment to improve the learners’ future outlook.

More research is needed to establish the support needs and appropriate interventions for learners with paraplegia in South African special schools. Furthermore, the impact of implementing peer support programmes within the school environment on the quality of life, health and educational outcomes of learners with paraplegia or SCI should be investigated.

## Conclusion

Formalised peer support through support groups and individual mentoring is an unmet need of learners with paraplegia in the participating South African special school. Physiotherapists should provide support, knowledge and advocacy for peer support programmes of learners with paraplegia in special schools at various levels. A well-structured, guided peer-support system could help to decrease pressure ulcers in the school setting.
